# Supplementary material for: Detection and Prediction of Macrophage Activation Syndrome in Still’s Disease
Source: J Clin Med. 2021 Dec 31;11(1):206. doi: 10.3390/jcm11010206 (PMC8745834; doi:10.3390/jcm11010206)
Supplement: Supplementary file 1 [file jcm-11-00206-s001.zip › jcm-1512057-supplementary.pdf]

Supplementary tables

Table S1. Comparison of clinical and biological characteristics of SD patients with and without MAS in adult and children.

**Characteristics**	*AOSD n = 128*			*SJIA n = 78*		
	**With MAS** *n* **= 11**	**Without MAS** *n* **= 117**	***p* value**	**With MAS** *n* **= 9**	**Without MAS** *n* **= 69**	***p* value**
Age at diagnosis, median (IQR), y	27.0 (25.0–54.0)	40 (30.0–55.0)	0.107	10.0 (4.75–14.2)	8.0 (4.0–11.8)	0.561
Sex (male), No. (%)	1 (9.09%)	47 (40.2%)	0.052	6 (66.7%)	32 (46.4%)	0.305
Classification criteria						
ILAR, No. (%)	NA	NA	NA	3/8 (37.5%)	24/ 52 (46.2%)	0.719
PReS, No. (%)	NA	NA	NA	3/7 (42.9%)	28/49 (57.1%)	0.688
Fautrel, No. (%)	9/10 (90.0%)	70/109 (64.2%)	0.161	NA	NA	NA
Yamaguchi, No. (%)	9/10 (90.0%)	63/109 (57.8%)	0.086	NA	NA	NA
Clinical features						
Fever, No. (%)	10/10 (100%)	108/125 (93.9%)	1.000	9 (100%)	57/63 (90.5%)	1.000
Skin rash, No. (%)	8/10 (80.0%)	78/115 (67.8%)	0.723	6 (66.7%)	42/59 (71.2%)	1.000
Arthralgia/arthritis, No. (%)	9/10 (90.0%)	31/98 (87.8%)	1.000	5/8 (62.5%)	61/66 (92.4%)	**0.037**
Splenomegaly, No. (%)	1/10 (10.0%)	14/94 (14.9%)	1.000	1 (11.1%)	5/46 (10.9%)	1.000
Hepatomegaly, No. (%)	3/10 (30.0%)	10/91 (11.0%)	0.118	3 (33.3%)	5/45 (11.1%)	0.118
Heart involvement, No. (%)	3/11 (27.3%)	25/113 (22.1%)	0.710	3/8 (37.5%)	7/57 (12.3%)	0.098
Digestive involvement, No. (%)	2/10 (20.0%)	19/111 (17.0%)	0.682	2 (22.2%)	11/57 (19.3%)	1.000
Neurologic involvement, No. (%)	2 (18.2%)	7/117 (5.98%)	0.173	2 (22.2%)	3 (4.35%)	0.099
Biological features						
WBCs, median (IQR), G/L	5.4 (3.5–13.4)	14.5 (9.9–18.5)	**0.004**	6.10 (4.4–14.3)	16.7 (14.0–21.0)	**0.013**
Hemoglobin, mean (SD), g/L	108 (14.4)	117 (17.5)	0.063	106 (26.4)	104 (13.6)	0.892
Platelets, median (IQR), G/L	175 (140–199)	326 (238–472)	**<0.001**	167 (122-358)	443 (365–556)	**0.001**
Serum ferritin, median (IQR), μg/L	15887 (6044–24684)	2760 (654–9803)	**0.046**	11000 (4464–109079)	582 (246–2830)	**0.001**
LDH, median (IQR), U/L	678 (527–1072)	340 (236-502)	**<0.001**	894 (573–1395)	347 (278–438)	**0.002**
ASAT, median (IQR), U/L	134 (95–186)	41 (25–68)	**<0.001**	126 (44–450)	28 (23–39)	**0.002**
ALAT, median (IQR), U/L	115 (86–175)	45 (21–86)	**0.001**	147 (36–291)	15 (10–24)	**0.004**
Fibrinogen, median (IQR), mmol/L	4.04 (1.50)	6.61 (2.03)	**<0.001**	2.62 (2.58)	5.65 (2.00)	**0.012**
PT, mean (SD), %	78.0 (69.0–84.5)	78.0 (69.0–94.5)	0.800	55.9 (18.6)	76.4 (15.2)	**0.028**
Triglycerides, median (IQR), mmol/L	2.90 (1.86–3.12)	1.53 (1.21–2.04)	**0.009**	2.20 (1.95–2.90)	1.40 (1.02–2.10)	**0.036**

ALAT, alanine-aminotransferase; AOSD, adult-onset Still's disease; ASAT, aspartate-aminotransferase; SD, standard deviation; ILAR, International League of Associations for Rheumatology; IQR, interquartile range; LDH, lactate dehydrogenase; MAS, macrophage activation syndrome; PReS, Pediatric Rheumatology European; PT, prothrombin time; SJIA, systemic-onset juvenile idiopathic arthritis; WBCs, white blood cells

Table S2. Comparison of SD patients with late-onset MAS and without MAS

Characteristics	Late-onset MAS<br>*n* = 8	Without MAS<br>*n* = 186	*p* value
Age at diagnosis median (IQR), y	12.0 (4.5–14.5)	29.0 (11.0–43.8)	**0.012**
Virus tests			
Positive CMV PCR, No. (%)	1/5 (20.0%)	5/48 (10.4%)	0.465
Positive EBV PCR, No. (%)	1/5 (20.0%)	10/51 (19.6%)	1.000
Clinical features			
Skin rash, No. (%)	6/7 (85.7%)	120/174 (69.0%)	0.677
Arthritis/arthralgia, No. (%)	6/7 (85.7%)	162/181 (89.5%)	0.551
Lung involvement, No. (%)	3/7 (42.9%)	32/171 (18.7%)	0.138
Heart involvement, No. (%)	3/7 (42.9%)	32/170 (18.8%)	0.140
Splenomegaly, No. (%)	1/6 (16.7%)	19/140 (13.6%)	0.594
Hepatomegaly, No. (%)	1/6 (16.7%)	15/136 (11.0%)	0.519
Biological features			
PT, median (IQR), %	62.5 (54.0–68.0)	78.0 (69.0–93.2)	**0.004**
ASAT, median (IQR), U/L	55 (31–94)	36 (24-–59)	0.359
ALAT, median (IQR), U/L	66 (22–78)	27 (15–62)	0.525
GGT, median (IQR), U/L	40 (16–100)	50 (22–120)	0.529
LDH, median (IQR), U/L	410 (339–653)	344 (242–484)	0.205
Disease course			
Monocyclic (systemic), No. (%)	1 (12.5%)	58/156 (37.2%)	0.260
Polycylic (systemic), No. (%)	5 (62.5%)	50/156 (32.1%)	0.119
Chronic (articular), No. (%)	2 (25.0%)	48/156 (30.8%)	1.000

ALAT, alanine-aminotransferase; ASAT, aspartate-aminotransferase; CMV, cytomegalovirus; EBV, Epstein Barr virus; GGT, gamma-glutamyltransferase; LDH, lactate dehydrogenase; MAS, macrophage activation syndrome; PCR, polymerase chain reaction; PT, prothrombin time
